# Nematofauna of *Bryconops* cf. *affinis* (Characiformes, Iguanodectidae) and *Saxatilia brasiliensis* (Cichliformes, Cichlidae) from the Munim River basin, Northeastern Brazil

**DOI:** 10.1590/S1984-29612024051

**Published:** 2024-09-16

**Authors:** Melissa Querido Cárdenas, Marciara Lopes Silva, Diego Carvalho Viana, Simone Chinicz Cohen, Felipe Polivanov Ottoni

**Affiliations:** 1 Laboratório de Helmintos Parasitos de Peixes, Instituto Oswaldo Cruz, Fundação Oswaldo Cruz – FIOCRUZ, Rio de Janeiro, RJ, Brasil; 2 Programa de Pós-graduação em Ciência Animal, Universidade Estadual do Maranhão – UEMA, São Luís, MA, Brasil; 3 Núcleo de Estudos Morfofisiológicos Avançados, Universidade Estadual da Região Tocantina do Maranhão – UEMASUL, Imperatriz, MA, Brasil; 4 Laboratório de Sistemática e Ecologia de Organismos Aquáticos, Centro de Ciências de Chapadinha, Universidade Federal do Maranhão – UFMA, Campus de Chapadinha, Chapadinha, MA, Brasil

**Keywords:** Nematoda, ichthyoparasitology, biodiversity, Saxatilia, Bryconops, helminths, Nematoda, ictioparasitologia, biodiversidade, Saxatilia, Bryconops, helmintos

## Abstract

Populations of freshwater species have been declining rapidly and species are becoming extinct. Thus, understanding freshwater species distribution, trends and patterns is required. The Munim River basin is situated in a region with a phytogeographic interface between the Amazon and Cerrado biomes. Although the Munim basin ichthyofauna is currently relatively well-known, data on its helminth fauna is scarce. The present study aimed to characterize the nematofauna of *Bryconops* cf. *affinis* (Günther) and *Saxatilia brasiliensis* (Bloch) from two different localities in the middle section of the Munim River, and thus to contribute to the knowledge of biodiversity in this region. Specimens of *Bryconops* cf. *affinis* were parasitized with the nematodes *Procamallanus* (*Spirocamallanus*) *krameri* (Petter, 1974) and “*Porrocaecum*-like” species (larvae) in both localities. *Saxatilia brasiliensis* presented the nematodes *P.* (*S*.) *krameri*, *Pseudoproleptus* sp. (larvae), *Cucullanus* sp. and *Procamallanus* sp. (larvae). *Procamallanus* (*S*.) *krameri* was found parasitizing *S*. *brasiliensis* only from the Feio stream. Morphometric data and parasitological parameters are given. The present study provides the first record of nematodes for *B.* cf. *affinis* and for *S. brasiliensis* contributing to the knowledge of the helminth fauna of freshwater fishes from locations that have not yet been studied, such as the Munim River basin.

## Resumo

As populações de espécies de água doce têm diminuído rapidamente e espécies estão sendo extintas. Portanto, é necessário compreender a distribuição, as tendências e os padrões das espécies de água doce. A bacia do rio Munim está situada em uma região com uma interface fitogeográfica entre os biomas da Amazônia e do Cerrado. Embora a ictiofauna da bacia do Munim seja relativamente conhecida atualmente, os dados sobre sua fauna de helmintos são escassos. O presente estudo teve como objetivo caracterizar a nematofauna de *Bryconops* cf. *affinis* (Günther) e *Saxatilia brasiliensis* (Bloch) de duas localidades diferentes na seção média do rio Munim e, assim, contribuir para o conhecimento da biodiversidade nessa região. Os espécimes de *B.* cf. *affinis* estavam parasitados pelos nematoides *Procamallanus* (*Spirocamallanus*) *krameri* e espécies semelhantes à *Porrocaecum* (larvas) em ambas as localidades. *Saxatilia brasiliensis* apresentou os nematoides *Procamallanus* (*S*.) *krameri*, *Pseudoproleptus* sp. (larvas de terceiro estágio), *Cucullanus* sp. e *Procamallanus* sp. (larvas). *Procamallanus* (*S*.) *krameri* foi encontrado parasitando *S*. *brasiliensis* apenas no riacho Feio. O presente estudo fornece o primeiro registro de nematoides para *B.* cf. *affinis* e para *S. brasiliensis*, contribuindo para o conhecimento da fauna de helmintos de peixes de água doce da bacia do rio Munim.

## Introduction

Global biodiversity is under threat due to human activities. Factors such as natural habitat destruction, overexploitation, invasive species, pollution and climate change are the main threats to biodiversity at a global level (Hogue & Breon, 2022). These factors affect not only free-living organisms, but also parasites (Marcogliese, 2023). Populations of freshwater species have been declining more rapidly and species are becoming extinct at alarmingly higher rates than in marine and terrestrial realms, a phenomenon known as the “*freshwater biodiversity crisis*” (Dudgeon et al., 2006; Darwall et al., 2018; Harrison et al., 2018; Reid et al., 2019; Albert et al., 2021; Tickner et al., 2020; Ottoni et al., 2023). Thus, understanding freshwater species distributions, trends and patterns is required in order to be able to draw up plans and implement appropriate measures for modifying the curves of extinction rates and population decline. These aims should be considered one of humanity’s highest priorities (Darwall et al., 2018; Albert et al., 2021; Tickner et al., 2020; Ottoni et al., 2023).

Currently, despite the importance of recording biodiversity, there is a taxonomic crisis, caused by a reduction in the numbers of new taxonomists that are being trained. This field of science has generated little interest among the new generations of researchers. Consequently, the species inventories of some taxonomic groups have declined (Simões & Robles, 2023). Despite the importance of parasites, it is known that there are many species that still need to be discovered and described before the inventory of extant parasite biodiversity can be considered complete (Jorge & Poulin, 2018; Carlson et al., 2020; Poulin et al., 2023).

The Munim River basin is located in the eastern region of the state of Maranhão (northeastern Brazil), occupying an area of approximately 16,000 km^2^, and it is one of the main river systems of Maranhão (Ribeiro et al., 2014; Oliveira et al., 2020; Vieira et al., 2023). This river basin is situated in a region with a phytogeographic interface between the Amazon and Cerrado biomes (IMESC, 2019; Vieira et al., 2023). It is important to emphasize that according to Myers et al. (2000), the Brazilian Cerrado is one of the world’s hotspots for biological diversity.

Despite the importance of the Munim River basin, few studies had been made regarding its ichthyofauna until the year 2023, when a comprehensive fish fauna inventory of this river basin was published by Vieira et al. (2023), recording a total of 123 fish species. Although the Munim River basin ichthyofauna is currently relatively well-known, data on its helminth fauna remains very scarce.

*Bryconops* cf. *affinis* (Günther) (Characiformes, Iguanodectidae) is a small-sized freshwater fish species, reaching around 12 cm long at the adult stage, and with benthopelagic habits. It is a common and ubiquitous species often found in streams in regions with a tropical climate, but more frequently encountered at low tide in creeks with swift-flowing waters. In addition, it has an herbivorous or insectivorous diet. Regarding its reproductive behavior, they spawn in schools among plants (Froese & Pauly, 2023).

*Saxatilia brasiliensis* (Bloch) (Cichliformes, Cichlidae) is a small to medium-sized pike fish species that occurs in the benthopelagic deep zone of freshwater bodies, with distribution in the hydrographic basins of northeastern Brazil (Fricke & Eschmeyer, 2024; Froese & Pauly, 2023). It feeds on fish, crustaceans and insects (Gurgel et al., 2005).

The Nematoda, a diverse phylum of animals, comprehends approximately 30,000 nematode species are formally recognized, yet the actual number is estimated to be around 500,000, with half of these species being parasitic, impacts host population dynamics. Nematodes represents the fifth most diverse metazoan phylum (Hodda, 2022a, b). This great diversity leads to significant challenges in understanding these parasites, mainly in taxonomy and systematics, essential for investigating complex topics such as life cycles, pathology, and host-parasite interactions (Moravec, 1998; Padial et al., 2010; Pereira & González-Solís, 2022).

The aim of the present study was to characterize the nematofauna of these two host species, from two different localities in the middle section of the Munim River, and thus to contribute to the knowledge of biodiversity in this region.

## Material and Methods

The fishes of the present study were collected from two localities: Feio Stream, located in the village of São José, in the municipality of Chapadinha (03º51'18.1”S 043º17'14.0”W and altitude of 11 m) (due to its low population density it has less anthropogenic activity), and Estrela Stream located in the municipality of Anapurus (03°40'15.6”S 043°7'9.7”W and altitude of 80 m) (a region strongly influenced by human activity). Both are in the eastern region of the State of Maranhão, northeastern Brazil, about 30 km apart in a straight line from each other, and their water bodies drain to the middle Munim River section.

For investigation of endohelminths, 64 specimens of *Bryconops* cf. *affinis* (32 from Feio Stream, São José village and 32 from Estrela Stream, Anapurus municipality) and 64 specimens of *Saxatilia brasiliensis* (32 from each locality), were obtained in the period between September 2021 and April 2022. The hosts were captured by using dip nets, trail nets, and cast nets, and were taken to the “Laboratório de Sistemática e Ecologia de Organismos Aquáticos (LASEOA)” of the“Universidade Federal do Maranhão (UFMA)”, where the specimens (fishes) were identified and euthanized by medullary section.

Posteriorly, the nematodes were collected from intestine and swimm bladder and washed in 0.65% NaCl solution, fixed in alcohol 70% and sent to the “Laboratório de Helmintos Parasitos de Peixes (Fiocruz)”, Rio de Janeiro. After that, the fish specimens were preserved in formalin (10%) and after 10-15 days moved to a 70% ethanol solution. All the fish material are housed at the “Coleção Ictiológica do Centro de Ciências Agrárias e Ambientais (CICCAA)” of the “Universidade Federal do Maranhão”, voucher numbers (CICCAA07121 – CICCAA07152).

For light microscopical examination (LM) the nematodes were cleared in lactophenol and observed using a Zeiss Axioscope 2 microscope with differential interference contrast (DIC), equipped with a camera lucida. All measurements are given in millimeters; range values are followed by means. To describe the parasitological parameters, data related to parasite as prevalence (P), intensity (I), mean intensity (MI) and range (R) were used according to Bush et al. (1997).

## Results

Among the 64 specimens of *Bryconops* cf. *affinis* examined, 17 were parasitized by nematodes (8 from the locality São José and 9 from Anapurus); while among the 64 specimens of *S. brasiliensis* examined, 20 were parasitized by this same parasite group (10 from São José and 10 from Anapurus).

Specimens of *Bryconops* cf. *affinis* were parasitized with the nematodes *Procamallanus* (*Spirocamallanus*) *krameri* (Petter, 1974) in the intestine and “*Porrocaecum*-like” species (third-stage larvae) in the swim bladder, in both localities. *Saxatilia brasiliensis* presented the nematodes *Procamallanus* (*S*.) *krameri*, *Pseudoproleptus* sp. (third-stage larvae), *Cucullanus* sp. and *Procamallanus* sp. (larvae), from the intestine. *Procamallanus* (*S*.) *krameri* was found parasitizing *S*. *brasiliensis* only from the Feio stream (São José). The mean abundance, prevalence, mean intensity and range of infection of the nematodes collected from both fish hosts are shown in Tables 1 and 2.

**Table 1:** Site of infection (SI), prevalence (P), mean intensity (MI), mean abundance (MA) and range of infection (R) of nematodes collected from *Bryconops* cf. *affinis* in Brazil.

**Sampling site**	**Parasite**	**SI**	**P (%)**	**MI**	**MA**	**R**
Feio stream, São José village, Chapadinha municipality	*Procamallanus* (*S*.) *krameri*	In	15.6	1.4 ± 1.41	0.125 ± 19.79	1-2
	*“Porrocaecum*-like species”	SB	15.6	4 ± 10,60	0.625 ± 8.48	2-8
Estrela stream, Anapurus municipality	*Procamallanus* (*S*.) *krameri*	In	15.6	1.6 ± 2.12	0.187 ± 18.38	1-2
	*“Porrocaecum*-like species”	SB	6.25	55 ± 190.92	8.59 ± 171.82	21-90

In: Intestine; SB: Swim bladder.

**Table 2:** Site of infection (SI), prevalence (P), mean intensity (MI), mean abundance (MA) and range of infection (R) of nematodes collected from *Saxatilia brasiliensis* in Brazil.

**Sampling site**	**Parasite**	**SI**	**P (%)**	**MI**	**MA**	**R**
Feio stream, São José village, Chapadinha municipality	*Procamallanus* (*S*.) *krameri*	In	3.12	2 ± 0.70	0.06 ± 21.21	2
	*Pseudoproleptus* sp.	In	15.62	3.2 ± 7.77	0.50 ± 11.31	2-5
	*Procamallanus* sp. (larva)	In	3.12	1	0.03 ± 21.92	1
	*Cucullanus* sp.	In	3.12	1	0.03 ± 21.92	1
Estrela stream, Anapurus municipality	*Procamallanus* sp. (larva)	In	3.12	3 ± 1.41	0.15 ± 19.09	2-3
	*Pseudoproleptus* sp.	In	18.75	1.5 ± 2.12	0.28 ± 16.26	1-2
	*Cucullanus* sp.	In	9.37	1.5 ± 1.41	0.18 ± 18.38	1-2

In: Intestine.

Considering that the nematode species found in the present study are already well described, only the main measurements with a brief description are presented here.

Family Camallanidae Railliet & Henry, 1915

Genus *Procamallanus* Baylis, 1923

**Figure 1:**
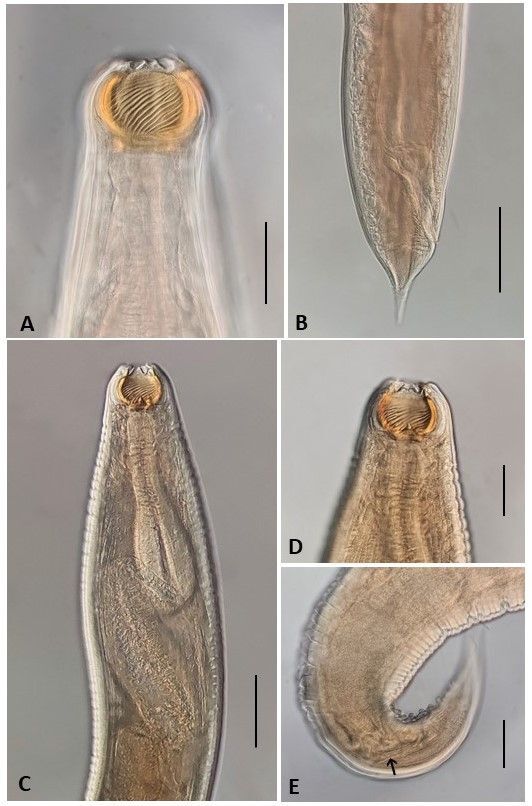
*Procamallanus* (*Spirocamallanus*) *krameri*. (A) Detail of anterior region of female, showing the buccal capsule withouth tooth-like protrusions in its bottom. Bar 0.1 mm; (B) Posterior region of female. Bar 0.15 mm; (C) Anterior region of male showing a buccal capsule with spiral thickenings absent in its anterior third. Bottom of buccal capsule presenting three elevated sclerotized protuberances (tooth-like protusions). Bar 0.12 mm; (D) Detail of buccal capsule showing one of tooth-like protusion on its bottom. Bar 0.06 mm; (E) Posterior region of male showing one of spicules (arrow). Bar 0.085 mm.

*Procamallanus* (*Spirocamallanus*) *krameri* (Figure 1)

Host: *Bryconops* cf. *affinis* and *Saxatilia brasiliensis*

Site of infection: Intestine

Medium-sized nematodes bearing an orange-brown buccal capsule with spiral ridges and a basal ring. Muscular esophagus shorter than glandular one; excretory pore situated posteriorly to nerve ring level. In males, spiral thickenings are absent in the anterior third, and there are three tooth-like protrusions at the bottom of the capsule. In females, the spiral thickenings cover the whole capsule, with absence of tooth-like protrusions.

Male (based on 6 specimens of *B.* cf. *affinis*): Body 2.00-5.27 (3.06) long by 0.17-0.25 (0.19) at the maximum width. Buccal capsule 0.06-0.07 (0.07) in length, including basal ring, by 0.08-0.10 (0.08) in maximum width, with spiral thickenings 11-19 (14) in number. Spiral thickenings limited to the middle third of surface, and the bottom of the capsule bears 3 tooth-like protrusions. Muscular esophagus 0.27-0.35 (0.29) in length; glandular esophagus 0.35-0.42 (0.42) in length. Nerve ring and excretory pore at 0.12-0.16 (0.15) and 0.18-0.34 (0.24) from anterior extremity, respectively. Posterior end of body provided with 10 pairs of caudal papillae (4 pairs of precloacal papillae and 6 pairs of postcloacal papillae). Two equal spicules of 0.07-0.20 (0.10) in length. Tail conical.

Female (based on 7 specimens of *B.* cf. *affinis*): Body 8.50-19.47 (15.5) long by 0.32 - 0.52 (0.47) at the maximum width. Buccal capsule 0.092 - 0.105 (0.098) in length, including basal ring, by 0.122 - 0.147 (0.131) in maximum width, with spiral thickenings 21-28 (24) in number. In females, the spiral thickenings cover the whole capsule and there are no basal teeth. Muscular esophagus 0.38 - 0.40 (0.39) in length; glandular esophagus 0.45-0.78 (0.62) in length. Nerve ring and excretory pore at 0.21-0.23 (0.22) and 0.22 - 0.41 (0.34) from anterior extremity, respectively. Vulva 2.05-9.67 (6.77) from anterior end. Distance from anus to posterior region 0.15 - 0.23 (0.18). Tail conical.

Male (based on 2 specimens of *Saxatilia brasiliensis*): Body 2.66 and 3.28 long by 0.160 and 0.170 wide. Buccal capsule 0.062 and 0.072 in length, including basal ring, by 0.065 and 0.067 in maximum width, with 12 spiral thickenings, limited to the middle third of the surface. Bottom of the capsule bearing 3 tooth-like protrusions. Muscular esophagus 0.257 and 0.275 in length; glandular esophagus 0.350 and 0.400 in length. Nerve ring and excretory pore at 0.125 and 0.150, and 0.25 from anterior extremity, respectively. Posterior end of body provided with 10 pairs of caudal papillae (4 pairs of precloacal papillae and 6 pairs of postcloacal papillae). Two equal spicules of 0.09 in length. Tail conical.

Remarks:

*Procamallanus* (*Spirocamallanus*) *krameri* was originally described as *Spirocamallanus krameri* by Petter (1974) from the intestine of *Hoplerythrinus unitaeniatus* (Spix & Agassiz) from French Guiana and was later reported from the same host in Venezuela (state of Barinas) and Brazil (state of Pará) (Moravec et al., 1997; Pinheiro et al., 2020, 2021). *Procamallanus* (*S*.) *krameri* is reported here from the Munim River Basin (state of Maranhão) for the first time, and *Bryconops* cf *affinis* and *Saxatilia brasiliensis* are new host records for this nematode.

This species is very similar to *Procamallanus* (*Spirocamallanus*) *inopinatus* but can differ regarding the structure of the buccal capsule. In *P*. (*S*). *krameri*, there is sexual dimorphism in this structure, such that spiral thickenings are absent from the anterior third to half of the capsule in males but cover the whole capsule in females; and in the males, there are three tooth-like protrusions at the bottom of the capsule. In *P*. (*S*.) *inopinatus*, the buccal capsules of both sexes are similar (the spiral thickenings cover two thirds of the buccal capsule and there are no tooth-like protrusions at the bottom).

**Figure 2:**
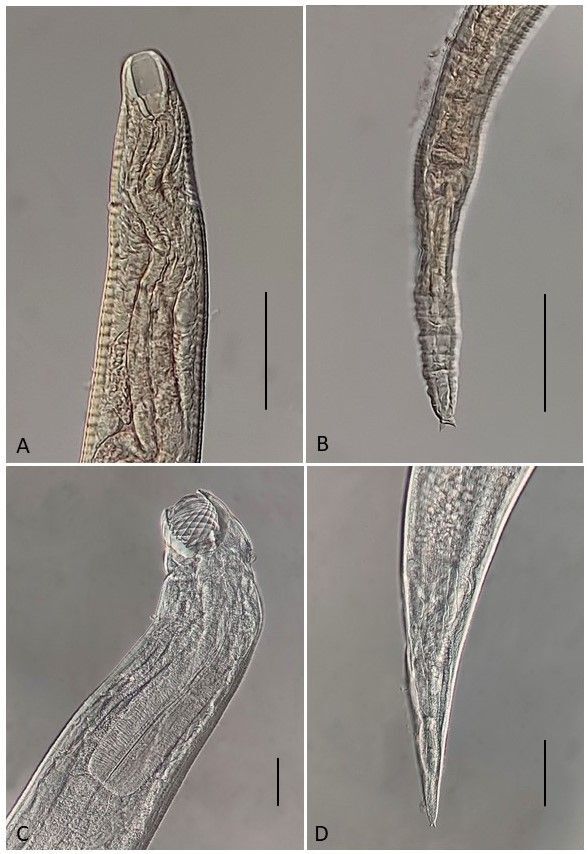
*Procamallanus* sp. (A) Third-stage larva bearing a smooth buccal capsule; (B) Third-stage larva with a conical tail bearing two terminal processes in its end; (C) Anterior end of fourth-stage larva with a buccal capsule with spiral ridges; (D) Tail of fourth-stage larva bearing two terminal processes in its end. Bars: 0.05 mm.

*Procamallanus* sp. (Figure 2)

Host: *Saxatilia brasiliensis*

Site of infection: Intestine

Larvae 3^rd^ stage (based on 1 specimen): Body 0.70 long by 0.05 at the maximum width. Buccal capsule 0.027 in length, by 0.017 in maximum width, without spiral thickenings. Muscular esophagus and glandular esophagus 0.135 and 0.125, respectively. Tail conical, bearing two terminal processes.

Larvae 4^th^ stage (based on 3 specimens): Body 1.95-2.30 (2.12) long by 0.10-0.14 (0.11) at the maximum width. Buccal capsule 0.045-0.050 (0.047) in length, by 0.057 (0.057) in maximum width, with 8-11 spiral thickenings. Muscular esophagus and glandular esophagus 0.242-0.250 (0.246) and 0.225-0.275 (0.250), respectively. Distance from nerve ring to anterior region 0.105-0.162 (0.133), and from anus to posterior region 0.115-0.145 (0.128). Tail conical, bearing two terminal processes.

Remarks:

Four specimens of *Procamallanus* sp. larvae were found parasitizing *Saxatilia brasiliensis* and one of them presented a smooth buccal capsule. Moravec et al. (1995) demonstrated that the third-stage larvae of *Procamallanus* (*S*.) *rebecae* do not present spiral ridges in the buccal capsule, similar to the specimen found here. Although *S. brasiliensis* is also parasitized by *P.* (*S*) *krameri*, it is not possible to affirm that they are conspecific.

Family Cystidicolidae Skrjabin, 1946

Genus *Pseudoproleptus* Khera, 1953

**Figure 3:**
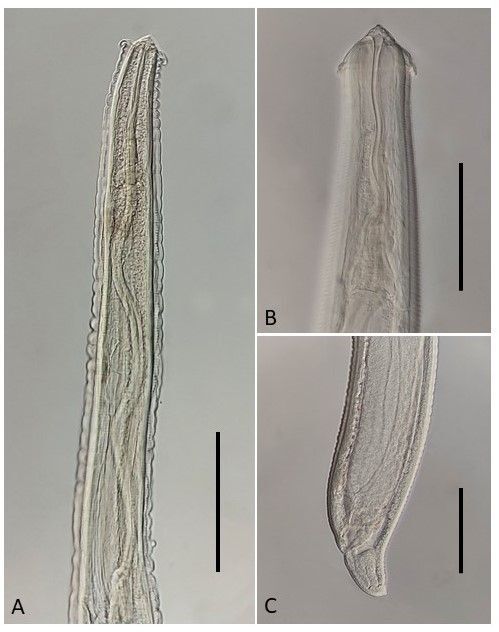
Third-stage larva of *Pseudoproleptus* sp. (A) Anterior region. Bar 0.20 mm; (B) Detail of anterior region showing the cephalic helmet-like cuticular structure. Bar 0.10 mm; (C) Detail of posterior region. Bar 0.24 mm.

*Pseudoproleptus* sp. (third-stage larvae) (Figure 3)

Host: *Saxatilia brasiliensis*

Larvae (based on 10 specimens): Body 9.26-22.00 (14.08) long by 0.11-0.19 (0.15) in maximum width. Anterior end of body with cephalic helmet-like cuticular structure 0.020-0.045 (0.036) long, 0.035-0.075 (0.062) in maximum width. Cephalic end rounded, with two pseudolabial protrusions. Vestibule with prostom 0.087-0.175 (0.143) long. Length of muscular esophagus 0.51-0.88 (0.70); length of glandular esophagus 1.65-3.90 (2.68); ratio between muscular and glandular parts of esophagus 1:2.99-5.09 (4.06); Nerve ring and excretory pore 0.157-0.235 (0.189) and 0.312-0.550 (0.431), respectively, from anterior extremity. Distance from anus to posterior region 0.09-0.16 (0.12). Tail conical, bearing a small knob-like terminal projection.

Remarks:

*Pseudoproleptus* sp. larvae were reported for the first time in Brazil in an Amazon River prawn, *Macrobrachium amazonicum* (Heller) (Decapoda, Crustacea) (Moravec & Santos, 2009). Melo et al. (2011) reported this nematode larva in *Satanoperca jurupari* (Cichliformes, Cichlidae) from the Guamá River, Pará. Subsequently, this nematode larva has been reported from different fishes in the Amazon region, mainly of the orders Cichliformes (Tavares-Dias et al., 2014, 2018, 2019; Pantoja et al., 2015; Pinheiro et al., 2019) and Characiformes (Morey & Malta 2018; Oliveira et al., 2018; Hoshino & Tavares-Dias, 2019; Morais et al., 2019). *Saxatilia brasiliensis* is a new host for this species, which is reported here for the first time in the Munim River basin. Although the life cycle of Cystidicolidae has not yet been well studied, it is known that freshwater crustaceans or aquatic insects are intermediate hosts for these nematodes (Moravec, 2007). Even though *Pseudoproleptus* sp. larvae have been reported in different fish species, some authors have stated that they are paratenic hosts (Melo et al., 2011; Morais et al., 2019). Further studies may contribute towards better understanding the life cycle of this nematode.

Family Cucullanidae Cobbold, 1864

**Figure 4:**
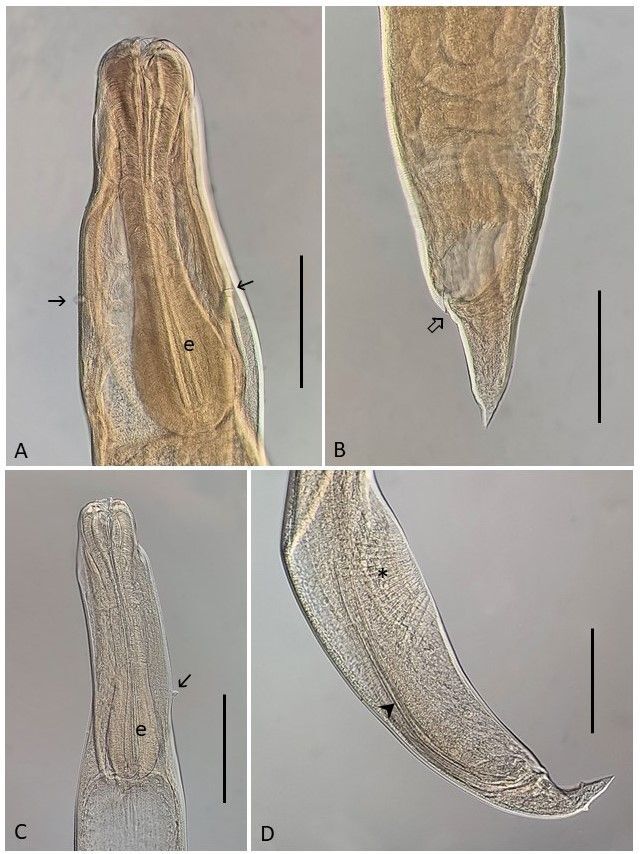
*Cucullanus* sp. (A) Anterior region of female showing the esophagus (e) and a pair of deirids (thin arrows); (B) Posterior region of female showing the anus (arrow); (C) Anterior region of female showing the esophagus (e) and one of the deirids (thin arrow); (D) Posterior region of male showing the spicules (arrow head) and the precloacal sucker (asterisk). Bars 0.2 mm.

*Cucullanus* sp. (Figure 4)

Host: *Saxatilia brasiliensis*

Site of infection: Intestine.

Male (based on 2 specimens): Body 3.20 and 3.80 in length by 0.20 and 0.30 in maximum width. Esophagus 0.52 and 0.53 in maximum length, accounting for 13-15% of whole-body length. Esophastome 0.14 and 0.15 long. Nerve ring and excretory pore situated at 0.185 and 0.210, and 0.30 from anterior extremity, respectively. Precloacal sucker 0.455 and 0.510 from posterior region. Spicules subequal with pointed distal end. Right spicule 0.437 and 0.410 and left spicule 0.437 and 0.487 long. Gubernaculum sclerotized, 0.067 long. Caudal papillae: 6 pairs of preanal, 2 pairs of adanal and 7 pairs of postanal papillae. Tail conical. Distance from cloaca to end of body 0.135.

Female (based on 5 specimens): Body 3.46-5.72 (4.36) long and 0.19-0.32 (0.26) in maximum wide. Esophagus 0.52-0.68 (0.59) long; esophastome 0.135-0.175 (0.153) long. Esophagus represents 15% (12-16%) of the body length. Distance of the nerve ring and excretory pore from the anterior extremity 0.165-0.237 (0.205) and 0.300-0.312 (0.306), respectively. Vulva postequatorial. Distance from vulva to the posterior extremity of the body 1.50-2.26 (1.70). Eggs 0.040-0.045 (0.042) long and 0.030-0.032 (0.031) wide. Tail conical, provided with a pair of lateral phasmids. Distance from anus to the end of body 0.115-0.187 (0.157).

Remarks:

Nematodes of the genus *Cucullanus* Müller, 1777 are parasites of marine, brackish-water and freshwater fish species around the world (Timi & Lanfranchi, 2006). In Brazil, 28 species of *Cucullanus* have been reported parasitizing freshwater, estuarine and marine fish species. Among these, only *Cucullanus tucunarensis* Lacerda, Takemoto, Marchiori, Martins & Pavanelli, 2015, and *Cucullanus opisthoporus* Pereira & Luque, 2017, were reported parasitizing cichlids (Lacerda et al., 2015; Pereira & Luque, 2017) and an unidentified species of *Cucullanus* was reported in *Cichla piquiti* (Lacerda et al., 2013; Yamada & Takemoto, 2013). This was the first record of these nematode species in *Saxatilia brasiliensis* in the Munim River basin. Further studies will be necessary in order to determine the species of this genus.

Subfamily Porrocaecinae Osche, 1958

**Figure 5:**
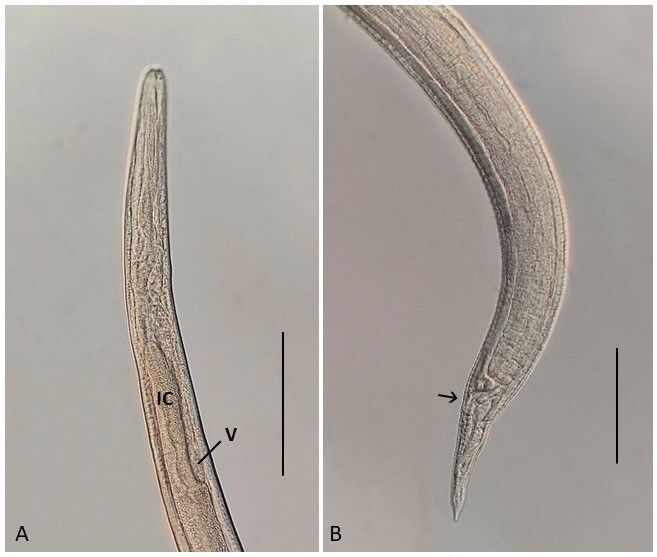
“*Porrocaecum*-like” species (third-stage larvae). (A) Anterior region showing the intestinal caeca (CI) and ventriculous (V). Bar 0.10 mm; (B) Posterior region showing the anus (arrow). Bar 0.08 mm.

“*Porrocaecum*-like” species (third-stage larvae) (Figure 5)

Host: *Bryconops* cf *affinis*

Larvae (based on 16 specimens): Body 1.42-2.15 (1.81) mm long by 0.05-0.07 (0.05) at the maximum width. Esophagus 0.20-0.40 (0.27) long, with a spherical ventriculus 0.022-0.037 (0.028) long and 0.015-0.020 (0.015) in width. Excretory pore and nerve ring situated at 0.085-0.175 (0.139) and 0.090-0.142 (0.116) from anterior end, respectively. Intestinal cecum 0.075-0.162 (0.106) long. Ventricular appendix absent. Distance from anus to posterior region 0.050-0.102 (0.083).

Remarks:

Martins & Onaka (2004) described larvae of *Porrocaecum* sp. in the swim bladder of the pacu (*Piaractus mesopotamicus* (Holmberg), a serrasalmid fish cultivated in Uberaba, state of Minas Gerais, Brazil, with the same morphological features as observed in the present material, and found at the same infection site. Recently, Pelegrini et al. (2021) reported *Porrocaecum* sp. larvae from the intestine of *Hoplosternum littorale* (Hancock) (Siluriformes, Callichthyidae), from the Batalha River, São Paulo, without description or figures. According to Luque et al. (2011), toxocarines are typically parasites of terrestrial higher vertebrates. Moreover, the Brazilian records of *Porrocaecum* spp. larvae are doubtful (Moravec, 1994, 1998) and all records of *Porrocaecum* spp. from fish are likely to be misidentifications of other ascarids, and are in reality probably taxa of anisakids. Thus, they can be considered to be “*Porrocaecum*-like” species (Luque et al., 2011).

Recently, Gu et al. (2023) described a new species of *Porrocaecum*, *P*. *moraveci*, and characterized the complete mitochondrial genomes of this species and of *P*. *reticulatum*, which are both parasites of *Circus aeruginosus* (Falconiformes: Accipitridae) in the Czech Republic. Their phylogenetic results demonstrated that *Porrocaecum* and *Toxocara* do not have an affinity. Consequently, the subfamily Porrocaecinae was resurrected, and the family Toxocaridae is also considered to be a subfamily, both belonging to Ascarididae. Further molecular studies may help elucidate the taxonomy of these larvae.

## Discussion

There are few studies about the nematode fauna of *Bryconops* spp.: Moravec & Thatcher (1997) reported the nematode *Procamallanus* (*Denticamallanus*) *dentatus* in *Bryconops alburnoides* Kner in the Urubu River, state of Amazonas, Brazil; Fujimoto et al. (2018) registered the nematode *Procamallanus* (*Spirocamallanus*) *inopinatus* in *B. melanurus* (Bloch) (Iguanodectidae) in the Caeté River, state of Pará; and Virgilio et al. (2022), in a study on the Juruá River, state of Acre, reported the nematodes *Contracaecum* sp., *Rondonia rondoni* Travassos, 1920 and *Travnema travnema* Pereira, 1938 in *Bryconops caudomaculatus* (Günther). The present study provides the first record of nematodes in *Bryconops* cf. *affinis* and *Saxatilia brasiliensis*. In addition, for the latter, there were no previous records of helminth species. The morphometric assessment on the nematodes studied here was in agreement with previously recorded data.

The trophic level of *Bryconops* cf. *affinis* and *Saxatilia brasiliensis* shows that these are species that can act as intermediate, paratenic or definitive hosts for several species of nematodes, thereby facilitating completion of their life cycles. This assertion is corroborated by the presence of parasite species, both in the adult form (such as *Procamallanus* (*Spirocamallanus*) *krameri* and *Cucullanus* sp.) and in larval forms (such as *Pseudoproleptus* sp., *Porrocaecum-*like species sp. and *Procamallanus* sp.), in the present study.

Among specimens of *Bryconops* cf. *affinis*, the highest prevalences were observed in *Procamallanus* (*S*.) *krameri* (15.6% in both localities) and *Porrocaecum*-like species (15.6% in the Feio stream), while *Pseudoproleptus* sp. (larva) presented the highest prevalence in *S. brasiliensis* (18.75% in the Estrela stream; 15.62% in the Feio stream). The prevalence of *Procamallanus* (*S*.) *krameri* observed in *Hoplerythrinus unitaeniatus* (Spix & Agassiz) (Erythrinidae) in the state of Pará was 75% (Pinheiro et al., 2021), i.e. higher than what was observed in the present study. *Porrocaecum*-like species presented prevalences of 17% in *P*. *mesopotamicus* in ponds in Minas Gerais (Martins & Onaka, 2004) and 18.87% in *Hoplosternum littorale* (Hancock) (Callichthyinae) in the Batalha River, state of São Paulo (Pelegrini et al., 2021), similar to what was observed for this species in the present study.

*Pseudoproleptus* sp. larvae have been reported from different fish species. The prevalence of this nematode in *S. brasiliensis* in the present study at both localities was similar to that found in other studies on different cichlid species, such as *Aequidens tetramerus* (Heckel) (Cichlidae) and *Chaetobranchus flavescens* Heckel (Cichlidae), both in the Igarapé Fortaleza basin, state of Amapá, with prevalences of 18.5% and 15.4%, respectively (Tavares-Dias et al., 2014, 2018). On the other hand, the prevalences found in the cichlids *Satanoperca jurupari* (Heckel) (Cichlidae) in the Guamá River, state of Pará, and *Mesonauta acora* (Castelnau) (Cichlidae) in the Igarapé Fortaleza basin, state of Amapá, were lower, 0.9% and 7.9%, respectively (Melo et al., 2011; Pantoja et al., 2015), than what was observed in the present study.

The highest mean intensity and mean abundance were found in relation to *Porrocaecum*-like species parasitizing *B*. cf. *affinis* in the Estrela stream (55 and 8.59, respectively). These figures are higher than what had been observed in previous studies on this species, with a mean intensity of 10 in *P*. *mesopotamicus* cultivated in Minas Gerais (Martins & Onaka, 2004) and a mean intensity of 2.30 and mean abundance of 0.43 in *Hoplosternum littorale* in the Tietê River, state of São Paulo (Pelegrini et al., 2021).

Among specimens of *S*. *brasiliensis*, the highest mean intensity and mean abundance were found in *Pseudoproleptus* sp. in fish in the Feio stream (3.2 and 0.5, respectively). These indexes were lower than what was previously observed in other cichlids, such as in *Astronotus ocellatus* (Agassiz) (Cichlidae) in the Tapajós River, state of Pará, with a mean intensity of 12.4 and mean abundance of 1.6 (Pinheiro et al., 2019). In *Aequidens tetramerus* and *C. flavescens* in the Igarapé Fortaleza basin, state of Amapá, the mean intensity of *Pseudoproleptus* sp. was 8.8 (in both) and the mean abundance was 1.6 and 1.4, respectively (Tavares-Dias et al., 2014, 2018). In *Laetacara curviceps* (Ahl), a cichlid species examined in the same locality, the mean intensity was 27.8 and the mean abundance was 16.7 (Pantoja et al., 2015). However, the indexes observed here were higher than that observed in *M. acora* in the Igarapé Fortaleza basin, which presented a mean intensity and mean abundance of 1.0 and 0.1, respectively (Tavares-Dias et al., 2019).

Negreiros et al. (2018) compared the helminth fauna of the Acre River (higher intensity of human activities) and Iaco River (lower intensity of human activity) and observed that the overall abundance and prevalence in the Acre River were higher than those observed in the Iaco River. Cavalcante et al. (2020) compared the helminth fauna of *Pimelodus blochii* Valenciennes (Pimelodidae) in the Acre River and Xapuri River, state of Acre, of which the latter was better preserved in terms of water quality. They observed higher abundance and prevalence in almost all species found in *P*. *blochii* in the Xapuri River, which suggested that anthropic action relating to the dumping of human sewage in the Acre River may be affecting the structure of the helminth community of *P*. *blochii*. Although in the present study some nematode species showed higher prevalence, mean abundance or mean intensity than others, the parasite indexes from fish in the Feio stream and Estrela stream were similar.

Brazil has a large number of freshwater ecosystems, many of which exhibit high diversity and endemism (Abell et al., 2008; Agostinho et al., 2005; Daga et al., 2016). However, many of these ecosystems have been disturbed by anthropic activities, which have threatened the maintenance of native biodiversity and ecosystems (Azevedo‐Santos et al., 2019; Albert et al., 2021; Ottoni et al., 2023). This scenario shows the importance of increasing the knowledge of the helminth fauna of freshwater fish in locations that have not yet been studied, such as the Munim River basin.
